# Rice straw-derived activated carbon/ZnO nanocomposite as a high-performance electrode for asymmetric supercapacitors

**DOI:** 10.1039/d6na00276e

**Published:** 2026-07-09

**Authors:** Asif Raza, Pravin MBS, Divya Rajendran, Zartasha Sarwar, Ranjithkumar Ravi, Nguyen Xuan Sang, Ramanathan Subramanian

**Affiliations:** a Department of Physics, University of South Africa Africa; b Department of Chemical Engineering and Materials Science, Amrita University Coimbatore India; c Medical Imaging Key Laboratory of Sichuan Province, Department of Oncology, Affiliated Hospital of North Sichuan Medical College Nanchong Sichuan China; d Department of Physics, The Women University Multan Pakistan; e Department of Physics, Faculty of Arts, Science, Commerce & Management, Karpagam Academy of Higher Education (Deemed to be University) Coimbatore India; f Atomic Molecular and Optical Physics Research Group, Institute for Advanced Study in Technology, Ton Duc Thang University Ho Chi Minh City Vietnam subramanianramanathan@tdtu.edu.vn; g Faculty of Electrical and Electronics Engineering, Ton Duc Thang University Ho Chi Minh City Vietnam; h Faculty of Applied Sciences, Ton Duc Thang University Ho Chi Minh City Vietnam

## Abstract

The synthesis of electrode materials from biomass waste has attracted considerable attention due to their low cost and high electrochemical performance in energy devices. Therefore, in this study, activated carbon was prepared from rice straw waste and composited with zinc oxide (ZnO) nanoparticles to enhance the electrochemical performance of an asymmetric supercapacitor. AC, ZnO, and AC-ZnO exhibit specific surface areas of 201, 255, and 578 m^2^ g^−1^, respectively, with average pore diameters of 1.37 nm, 1.73 nm, and 2.45 nm, respectively. The AC-ZnO composite exhibited a higher specific capacitance of 244 F g^−1^, compared with 206 F g^−1^ for ZnO and 137 F g^−1^ for AC at a current density of 0.5 A g^−1^. The charge-transfer resistance (*R*_ct_) values for AC, ZnO, and AC-ZnO were 0.050, 0.061, and 0.035 Ω, respectively. After 10 000 charge–discharge cycles, the corresponding capacitance retentions were 83.5%, 75.3%, and 89.9%, respectively. The AC-ZnO composite exhibits a hybrid capacitive behavior due to the electrical double-layer capacitor behavior of AC and the pseudocapacitive behavior of ZnO. The specific capacitance, energy density, and power density of the assembled device are 149 F g^−1^, 20.66 Wh kg^−1^, and 500 W kg^−1^, at 1 A g^−1^. Moreover, the assembled device exhibited 97% capacitance retention and 100% coulombic efficiency after 5000 charge–discharge cycles.

## Abstract 2

The synthesis of electrode materials from biomass waste has attracted considerable attention due to their low cost and high electrochemical performance in energy devices.

## Graphical abstract

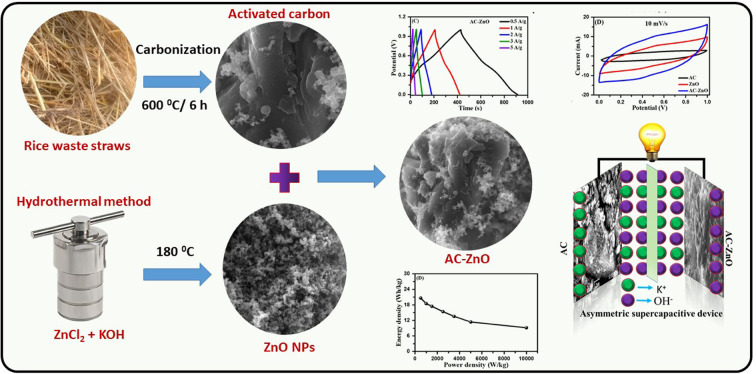

## 1. Introduction

Agricultural biomass wastes (ABWs), such as rice husks, rice straw, wheat straw, and date seeds, are generated in large quantities worldwide and have become a significant environmental concern due to improper disposal practices and open-field burning.^1^ The uncontrolled combustion of these residues releases large amounts of greenhouse gases and particulate matter, causing serious environmental pollution and adverse impacts on ecosystems and human health.^2^ Therefore, converting agricultural biomass wastes into value-added functional materials represents an effective strategy for sustainable waste management and environmental protection.^3–8^ Carbon-based materials are widely used as supercapacitor electrodes because of their high electrical conductivity, large surface area, excellent chemical stability, and long cycle life.^9^ However, conventional carbon materials such as graphene and carbon nanotubes often involve expensive synthesis procedures, limiting their large-scale application.^10^ In contrast, activated carbon derived from biomass waste is an attractive alternative due to its low cost, abundant availability, and environmentally friendly synthesis.

Nevertheless, the electrochemical performance of biomass-derived activated carbon is often limited by insufficient electrical conductivity and limited active surface sites. Various strategies have been developed to enhance its performance, including heteroatom doping and metal oxide incorporation.^11,12^ Heteroatom doping can improve electrical conductivity, surface polarity, wettability, and ion diffusion kinetics, thereby enhancing electrochemical performance.^13,14^ Furthermore, integrating metal oxides with carbon materials can introduce additional pseudocapacitive behavior and improve charge storage capacity.^15–17^ Among various metal oxides, zinc oxide (ZnO) has attracted considerable interest because of its moderate electrical conductivity, chemical stability, and pseudocapacitive properties. ZnO can serve as an effective component of carbon-based composites, facilitating electron transport and enhancing electrochemical activity.^18,19^ The incorporation of ZnO nanoparticles into carbon matrices can also generate oxygen vacancies and improve surface wettability, which promotes faster ion transport and improved electrochemical performance.^20–22^

Several studies have reported the development of ZnO–carbon composite materials for supercapacitor applications. For instance, Mohamed *et al.* used a one-step hydrothermal method to prepare ZnO-AC materials and measured their electrochemical performance in alkaline (KOH) and acidic (H_2_SO_4_) electrolyte solutions. The addition of ZnO to AC changed the chemical composition, resulting in improved electrochemical properties of the ZnO-AC materials.^23,24^ Zhang *et al.* developed a ZnO/CNT material, whose surface morphology changed from spherical to a lily-like shape by optimizing the preparation time.^25^ Xiao *et al.* successfully prepared an CS@ZnO core–shell structure, which exhibited a specific capacitance of 630 F g^−1^ at 2 A g^−1^.^24^ Saka *et al.* prepared AC from pomegranate peel waste and then doped it with nitrogen and ZnO nanoparticles. The composite materials exhibited a specific capacitance of 262 F g^−1^ and 86% cycle life after 5000 cycles.^26^ Madhu *et al.* reported the preparation of AC from sugarcane bagasse and decorated ZnO on its surface.^27^ The ZnO-AC material demonstrated superior electrochemical performance. Huang *et al.* reported the electrochemical performance of NCNF/BC@ZO/NiCo-LDH composites with a specific capacitance of 697 F g^−1^ and cycling stability of 80% after 10 000 cycles.^28^ Therefore, it is necessary to study the ZnO doping effects to improve the electrochemical performance of AC materials. Although several ZnO–carbon composites have been reported for supercapacitor applications, the use of rice straw waste as a precursor for activated carbon and its integration with hydrothermally synthesized ZnO nanoparticles remain relatively unexplored. Therefore, this work develops a low-cost and sustainable AC-ZnO nanocomposite derived from rice straw waste deposited on a graphite sheet current collector. This study provides an environmentally friendly and economically viable approach for converting agricultural waste into high-performance electrode materials for supercapacitor applications.

In this study, activated carbon was prepared from rice straw waste using a simple pyrolysis method, while ZnO nanoparticles were synthesized through a hydrothermal process and subsequently incorporated into the activated carbon matrix to enhance the surface properties and electrochemical performance of the composite electrode. Graphite sheets were employed as the current collector owing to their high electrical conductivity, chemical inertness, and excellent electrochemical stability. The graphitic surface possesses defect sites and surface functional groups that can serve as nucleation centers for ZnO growth and facilitate strong adhesion of the deposited active material. Additionally, the layered structure of graphite provides continuous electron-conduction pathways and good interfacial contact, thereby improving charge-transfer efficiency and long-term cycling stability of the electrode. The resulting AC-ZnO nanocomposite exhibits a high specific capacitance of 244 F g^−1^ at a current density of 0.5 A g^−1^ with excellent cycling stability. Furthermore, the assembled asymmetric supercapacitor device demonstrates a specific capacitance of 149 F g^−1^, an energy density of 20.66 Wh kg^−1^, and a power density of 500 W kg^−1^ at 1 A g^−1^ with 97% capacitance retention after 5000 cycles.

## 2. Experimental

### 2.1 Materials

Sulfuric acid (H_2_SO_4_) (98%), potassium hydroxide (KOH) (≥85%), polyvinylidene fluoride (PVDF), and zinc chloride (ZnCl_2_, 98%) were purchased from Sigma-Aldrich and used as received. Solvents such as ethanol, dimethylformamide (DMF), and *N*-methyl-2-pyrrolidone (NMP) were purchased from Sigma-Aldrich. Rice straw waste (RSW) was collected from agricultural land in Tamil Nadu, India. Double-distilled water (DD) was used throughout the experiments. Graphite sheets (0.2 mm thickness, purity >99%) were purchased from Sigma-Aldrich (USA) and used as the current collector without further modification.

### 2.2 Preparation of RSW-derived carbon

The collected RSW was washed several times with distilled water, air-dried, and then cut into small pieces. These small pieces were then carbonized in a tubular furnace at 600 °C for 6 h under an inert atmosphere. After the reaction was complete, the powdered form of RSW carbon was obtained and sealed in an airtight container.

### 2.3 Synthesis of zinc oxide (ZnO) nanoparticles

**Fig. 1:**
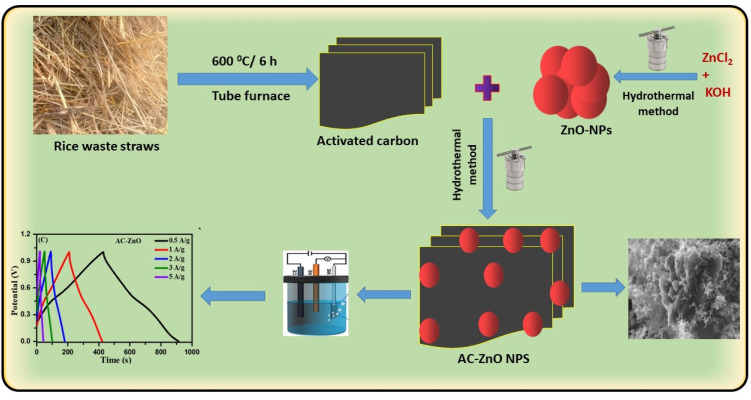
Schematic representation of the preparation of AC-ZnO composite materials.

The ZnO nanoparticles were synthesized using the following procedure. A 0.4 M ZnCl_2_ solution was prepared by using double-distilled water (DD) as solvent, then 0.8 M KOH solution was slowly added to maintain a Zn^2+^ : OH^−^ molar ratio of 1 : 2, and the mixture was continuously stirred for 2 h. The mixture was then transferred to a Teflon-lined autoclave and maintained at 180 °C for 24 h (Fig. 1). The mixture was then filtered through Whatman filter paper. The precipitate was then collected and washed several times with DD water and an ethanol solution, and dried to obtain the final solid product.

### 2.4 Synthesis of AC-ZnO nanocomposites

The AC-ZnO nanocomposites were synthesized from the following procedure: ZnO-NPs solution was prepared by DD water, and it was mixed with a dispersed form of RSW (calculated amount) with a 2 : 1 mass ratio by using ethanol as a solvent, then this solution mixture was subjected to magnetic stirring for 12 h (Fig. 1). The mixture was then transferred to a Teflon autoclave and heated at 180 °C for 24 h. The mixture was then filtered through a Whatman filter paper. Then, this filtrate was collected and washed several times with DD water and an ethanol solution. The product was dried at 80 °C for 5 h to obtain the powder form of the AC-ZnO nanocomposite.

### 2.5 Characteristics of synthesized materials

X-ray diffraction (XRD) patterns were recorded using a Shimadzu XRD 6000 (Japan) instrument with a Cu Kα radiation source and 2*θ* = 10–80°. Surface morphology of the synthesized materials was confirmed by a transmission electron microscope (TEM) JEM-2100 (Japan) instrument and a scanning electron microscope (SEM) JEM-2100 (Japan). Fourier transform infrared spectroscopy (FT-IR) was used to confirm the functional groups of the synthesized compound by using a Nicolet-6700 (USA) instrument over the wavenumber range of 4000–400 cm^−1^. Raman spectra were recorded using a confocal Raman spectrometer (Witec, Germany). Brunauer–Emmett–Teller analysis (BET) was used to measure the surface area of the prepared electrode material. The X-ray photoelectron spectra were analyzed by XPS (ESCA 5800, Japan) with an Al K_α_ X-ray source at 100 W in a vacuum pressure of 10^−7^ Pa.

### 2.6 Electrochemical performance

The working electrode was prepared by mixing the synthesized powder materials (AC, ZnO, or AC-ZnO) with carbon black and polyvinylidene fluoride (PVDF) in an 8 : 1 : 1 ratio in NMP by mass on a graphite sheet. Graphite sheets with dimensions of approximately 1 × 1 cm^2^ and a thickness of 0.2 mm were used as the current collector. Before coating, the graphite sheets were cleaned with ethanol and deionized water to remove surface contaminants and then dried at room temperature. The active material slurry was uniformly deposited onto the graphite sheet, resulting in an active material loading of approximately 2.2 mg cm^−2^. All electrodes were prepared using the same fabrication protocol, slurry composition, and drying conditions to ensure consistency and reproducibility of the electrochemical measurements. All electrochemical measurements were performed using three independently prepared electrodes, and the results are reported as mean ± standard deviation.

Electrochemical measurements were performed using a CHI-660C electrochemical workstation in a three-electrode configuration with 1 M H_2_SO_4_ electrolyte, a platinum wire counter electrode, an Ag/AgCl reference electrode, and a modified graphite sheet as the working electrode. The 1 M H_2_SO_4_ electrolyte was selected due to its high ionic conductivity and rapid proton transport, which enhance charge-transfer kinetics and electrochemical performance.^29–31^ The electrochemical tests were performed at room temperature (25 ± 1 °C). Cyclic voltammetry (CV) was conducted at different scan rates, in the 20–100 mV s^−1^ range, in the potential window of 0–1.0 V. The galvanostatic charge–discharge (GCD) measurements were carried out at current densities of 0.5, 1, 2, 3, and 5 A g^−1^, and the impedance measurements were carried out in a frequency range from 0.01 Hz to 100 kHz. The specific capacitance (*C*_s_), energy density (*E*_d_), and power density (*P*_d_) of the prepared electrodes were measured using the following equations.^32–35^1
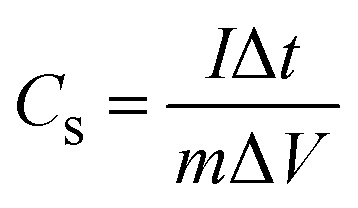
2
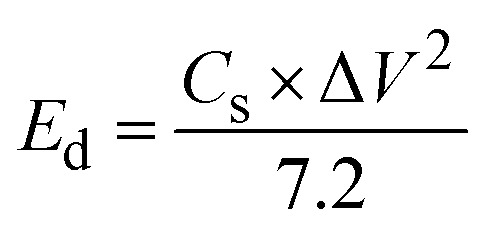
3
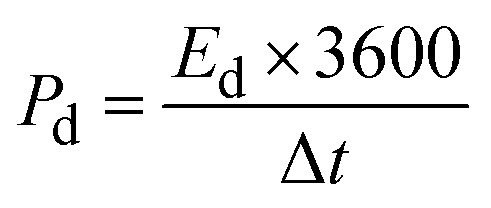
where *I* is the discharge current (A), Δ*t* is the discharge time (s), *m* is the mass of the active material (g), and Δ*V* is the potential window (V).

## 3. Results and discussion

### 3.1 Physical properties

**Fig. 2:**
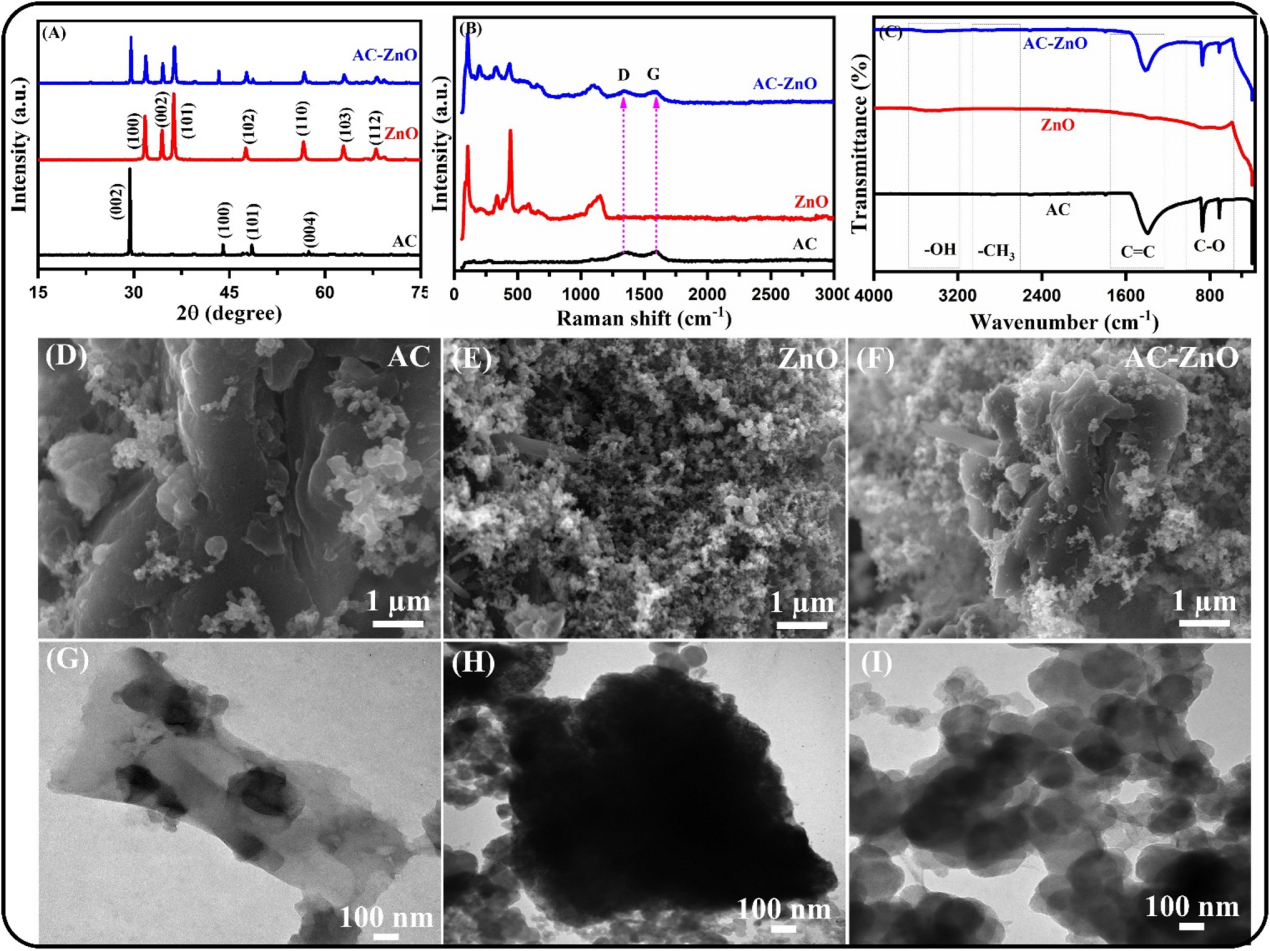
(A) XRD patterns and (B) vibration modes of AC, ZnO, and AC-ZnO composite, (C) chemical functional groups in AC, ZnO, and AC-ZnO composite, SEM images of (D) AC, (E) ZnO, and (F) AC-ZnO and microstructural properties of (G) AC, (H) ZnO, and (I) AC-ZnO composite.

The crystalline quality of the AC, ZnO, and AC-ZnO composite was evaluated using XRD analysis within a 2*θ* range of 10–80°. Fig. 2A displays the XRD patterns for AC (black line), ZnO (red line), and the AC-ZnO (blue line) composite. The broad diffraction peaks at 29.39°, 43.99°, 48.61°, and 57.47°, corresponding to graphitic carbon planes, indicate partially ordered turbostratic carbon structures.^36–38^ The first three peaks correspond to graphitic (sp^2^) carbon structures, while the broad peak at a higher angle indicates disordered carbon domains typical of turbostratic carbon structures,^39^ similar to AC. These peaks also reflect the turbostratic nature of AC, derived from randomly stacked carbon layers. This turbostratic structure confers a high surface area and more active sites on AC, thereby enhancing ion adsorption and electrochemical performance. The ZnO nanoparticles exhibit three strong peaks at 31.76°, 34.44°, and 36.27°, assigned to the (100), (002), and (101) planes, respectively, with additional weaker peaks at 47.63°, 56.62°, 62.94°, and 67.98°, corresponding to the (102), (110), (103), and (112) planes of the hexagonal wurtzite structure of ZnO [JCPDS No. 79-0207, 36-1451]. The high-intensity peaks indicate high crystallinity and purity of ZnO-NPs. The presence of these diffraction peaks at specific angles aligns with previous studies.^40,41^ In the case of the AC-ZnO composite, the diffraction peaks of both AC and ZnO shift to higher angles after forming the composite. The peaks become broader and less intense, indicating a reduction in crystallite size and crystallinity in the AC-ZnO composite. However, the ZnO peaks remain visible, demonstrating stability after hybridization with AC. Several factors can explain the decrease in peak intensity: (i) uniform dispersion of ZnO-NPs on the AC surface suppresses crystal growth, reducing crystallite size and causing broader, less intense peaks; (ii) the addition of AC may allow diffusion into the ZnO lattice, creating structural defects, lattice distortions, and micro-strains that diminish peak intensity; (iii) interactions at the interface between AC and ZnO introduce defects, further affecting the structure and reducing peak intensity. Additionally, the combination of crystalline ZnO and the amorphous AC matrix diminishes the overall peak intensity. The mismatch in ionic radii between Zn and C causes structural distortions. Nucleation between these materials suppresses the growth of crystallite size.

The confirmation of reducing crystallite size (*C*_s_) and *d*-spacing with increasing dislocation density (*δ*) and strain (*ε*) is validated by calculations using the following equations.^42–44^4
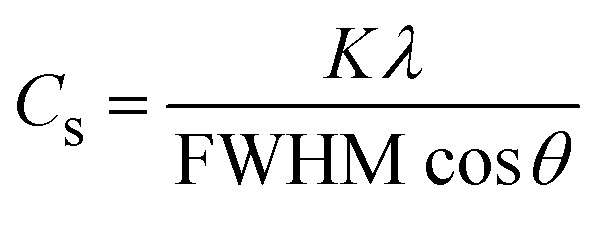
5
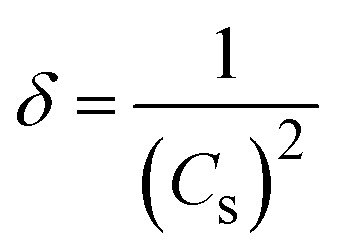
6
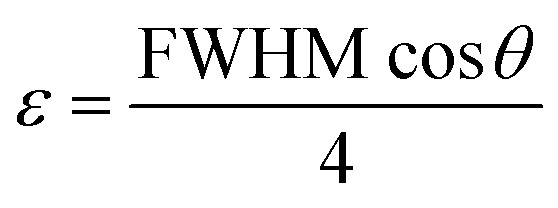
7
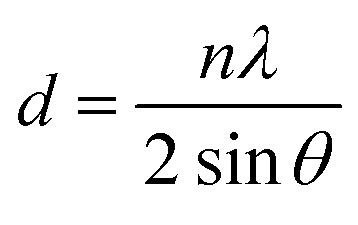
where *K* = shape factor (0.89), *λ* = X-ray wavelength (0.154 nm), *β* = FWHM (radians) and *θ* = Bragg angle. The highly intense (002) and (100) peaks show the preferential growth along *c* and *a* orientations of the AC and ZnO phases. The *C*_s_ value of the (002) plane is 60.59 nm and 54.88 nm for AC and the AC-ZnO composite, respectively [Table 1]. The (100) plane shows *C*_s_ values of 34.02 nm and 32.67 nm for ZnO and the AC-ZnO composite, respectively. The addition of AC to ZnO suppresses ZnO peaks, resulting in a higher FWHM and, consequently, a lower *C*_s_ value. A decreasing *C*_s_ value is beneficial for maximizing surface area and porosity, thereby improving interactions at ion-active sites during electrochemical performance.^45^

**Table 1:** Structural parameters of AC, ZnO, AC-ZnO composite

Sample	Plane (*h k l*)	2*θ* (°)	FWHM (°)	*C*_s_ (nm)	*δ* (10^−4^ nm^−2^)	*ε* (10^−2^)	*d*-Spacing (Å)
AC	(002)	29.41	0.13	60.59	2.72	3.24	3.10
	(100)	44.00	0.13	65.15	2.35	3.02	2.05
	(101)	48.54	0.19	45.34	4.86	4.33	1.87
	(004)	57.46	0.21	42.64	5.49	4.60	1.60
ZnO	(100)	31.78	0.24	34.02	8.64	5.77	2.81
	(002)	34.44	0.23	35.74	7.83	5.49	2.60
	(101)	36.28	0.26	31.78	9.89	6.17	2.47
	(102)	47.58	0.31	27.68	13.04	7.09	1.91
	(110)	56.63	0.35	25.48	15.39	7.70	1.62
	(103)	62.89	0.39	23.60	17.95	8.32	1.47
	(112)	67.98	0.44	21.53	21.57	9.12	1.37
AC-ZnO	(002)	29.58	0.15	54.88	3.32	3.57	3.02
	(100)	31.90	0.25	32.67	9.36	6.01	2.80
	(002)	34.56	0.22	37.38	7.15	5.25	2.59
	(101)	36.40	0.28	29.52	11.47	6.64	2.46
	(100)	43.33	0.07	115.76	0.74	1.69	2.08
	(102)	47.69	0.31	27.69	13.03	7.08	1.91
	(101)	48.71	0.21	41.05	5.93	4.78	1.86
	(110)	56.75	0.35	25.50	15.37	7.69	1.62
	(103)	63.02	0.41	22.46	19.81	8.74	1.47
	(112)	68.12	0.45	21.06	22.53	9.32	1.37

The *δ* value of (002) is increased from 2.72 × 10^−4^ nm^−2^ to 3.32 × 10^−4^ nm^−2^ for AC addition in ZnO. Meanwhile, the *δ* value of (100) is increased from 8.63 × 10^−4^ nm^−2^ to 9.36 × 10^−4^ nm^−2^ with AC-ZnO composite formation. Similarly, the *ε* value of (002) is increased from 2.72 × 10^−4^ nm^−2^ and 3.32 × 10^−4^ nm^−2^ for the AC-ZnO composite. However, the *d*-spacing values are decreased from 3.03 Å and 3.01 Å for (002), showing the addition of AC in the ZnO lattice. The addition of AC creates structural disorder in the ZnO lattice, as confirmed by increasing *δ* and *ε* values with decreasing *C*_s_ and *d*-spacing values. Variations in these structural parameters are conducive to improved electrochemical performance in AC-ZnO composite materials.

Fig. 2B displays the Raman vibrational modes of AC, ZnO, and the AC-ZnO composite material. The AC shows Raman bands at 1337 cm^−1^ and 1596 cm^−1^, corresponding to D and G bands. The first band indicates defects and disorder, while the second band reflects graphitic order in the AC.^46,47^ ZnO exhibits vibrational peaks at 102 cm^−1^, 333 cm^−1^, 382 cm^−1^, 445 cm^−1^, 585 cm^−1^, and 1140 cm^−1^.^48,49^ The characteristic peaks at 102 cm^−1^ and 445 cm^−1^ represent the *E*_2_ (low) and *E*_2_ (high) modes of ZnO-NPs. The peak at 333 cm^−1^ is also related to these high- and low-frequency second-order vibrational modes. The peak at 382 cm^−1^ indicates the crystallinity of the hexagonal wurtzite structure of ZnO and demonstrates the A1TO mode as well as the *E*_2_ high optical vibrational modes.^50,51^ A low-intensity peak at 585 cm^−1^ corresponds to the A1LO and E1LO modes of ZnO-NPs. Additionally, a broad peak at 1140 cm^−1^ is due to a multiphonon process.^52^ These vibrational modes of ZnO become less intense and broader upon the addition of AC. They are also redshifted due to structural defects induced by carbon diffusion into the ZnO lattice. These Raman findings are fully consistent with the XRD results.

The different functional groups of AC, ZnO, and the AC-ZnO composite are identified by FTIR analysis. Fig. 2C shows the FTIR spectra of AC, ZnO, and the AC-ZnO composite within the range of 500–4000 cm^−1^. The AC material displays minor absorption bands around ∼3400 to 3500 cm^−1^ and 2880–2900 cm^−1^, which are due to the stretching of O–H and C–H bonds, respectively.^53–59^ The presence of these minor bands results from oxygenated and lignocellulose species in the AC material. The strong absorption bands at approximately 1575 to 1600 cm^−1^ are attributed to C=C stretching of aromatic carbon domains, while weaker bands at 1100 to 1200 cm^−1^ correspond to C–O stretching from cellulose and hemicellulose groups.^60,61^ ZnO shows bands at 3437, 1630, 1533, 1378, 936, and 704 cm^−1^, indicating the presence of various functional groups.^62–64^ The minor peak at 3437 cm^−1^ confirms hydroxyl (OH) group stretching vibrations. Peaks at 1630 and 1533 cm^−1^ correspond to the C=O amide I and amide II groups—a single peak at 1378 cm^−1^ results from –C–H bending vibrations. The peaks at 936 and 704 cm^−1^ are due to bending vibrations of the =C–H group. In the AC-ZnO composite, similar absorption peaks are observed, confirming the purity of the product. The intensity of the O–H and C–H peaks decreases compared to AC, indicating significant interactions between ZnO-NPs and oxygen-containing functional groups on the AC surface. Slight shifts in these peaks are also observed, attributed to interactions between O–H and C–H groups forming a Zn–O–C linkage.^65–67^ ZnO interacts with various AC functional groups such as hydroxyl, carboxyl, and ether. Additionally, the vibration band of Zn–O becomes more prominent in the AC-ZnO composite, demonstrating the incorporation of ZnO-NPs into the carbon lattice. These interfacial bonds can facilitate rapid charge transfer and enhance structural stability, which are highly beneficial for electrochemical performance.

SEM and TEM analyses are used to investigate the surface morphology and microstructural properties of AC, ZnO, and the AC-ZnO composite, as displayed in Fig. 2D–I. The irregular and rough surface with layered morphology is revealed by AC (Fig. 2D). The surface morphology is dense due to the stacking of layers. The stacked layers are interconnected, exhibiting a porous structure developed during activation. These layers provide a large surface area and active sites for maximum ion interactions and charge storage during the electrochemical process, whereas densely packed and agglomerated ZnO-NPs are observed for pure ZnO (Fig. 2E). The uniform granular morphology with clusters of nanoparticles is clearly visible. The ZnO-NPs show well-defined boundaries, confirming good crystallinity in addition to their agglomeration. When ZnO-NPs are added to AC, the surface morphology is distinctly different (Fig. 2F). The ZnO-NPs are uniformly distributed over the large and porous surface of AC. The composite formation of AC-ZnO reduces the NPs' agglomeration. The carbon matrix inhibits the growth and clustering of ZnO-NPs. The intimate interfacial contact between ZnO and AC facilitates the formation of a conductive network and enhances structural stability, thereby benefiting electrochemical performance.

TEM analysis further confirms the amorphous nature of AC. The carbon layers are extremely thin and partially transparent with irregular edges (Fig. 2G). The absence of well-defined lattice fringes confirms the disordered carbon domains, while ZnO shows dense and dark regions, indicating aggregation of ZnO NPs (Fig. 2H). For the AC-ZnO composite (Fig. 2I), ZnO NPs are dark, uniformly embedded, and well-dispersed within the lighter carbon matrix. ZnO nanoparticles are uniformly anchored on the AC surface, indicating strong interfacial interactions between ZnO and the carbon matrix. The even dispersion of ZnO NPs in the carbon matrix reduces ion diffusion pathways and enhances the charge transfer rate in the AC-ZnO composite. The combined SEM and TEM results clearly demonstrate that AC acts as an effective support, stabilizing ZnO NPs, preventing severe agglomeration, and forming a synergistic composite structure, thereby improving electrochemical performance.

### 3.2 BET analysis

**Fig. 3:**
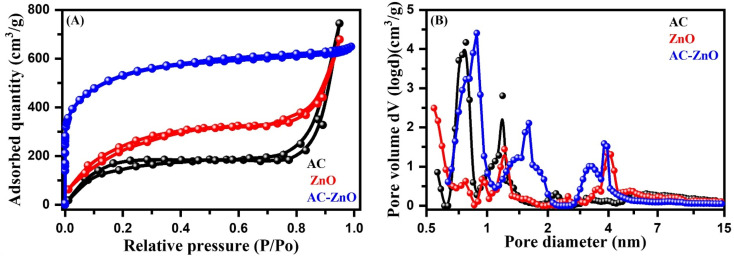
(A) N_2_ adsorption/desorption isotherms and (B) porosity of AC, ZnO, and AC-ZnO composite.

BET analysis was used to determine the surface area and porosity of AC, ZnO, and the AC-ZnO composite, as shown in Fig. 3. All these materials display a type IV isotherm with a hysteresis loop, indicating a mesoporous structure (Fig. 3A). ZnO exhibits a moderate surface area (∼255 m^2^ g^−1^), while the AC-ZnO composite shows a significantly higher surface area of 578 m^2^ g^−1^ due to the synergistic interaction between ZnO nanoparticles and porous carbon. This large surface area results from the uniform dispersion of ZnO NPs within the AC matrix. The dispersion of ZnO NPs enhances pore development, creating a porous network in the AC-ZnO composite. The increased surface area results from the porous carbon framework and the uniform dispersion of ZnO nanoparticles within the AC matrix, which promotes pore formation *via* lattice distortion caused by the different ionic radii of Zn and C atoms. The surface area of this composite exceeds that of similar materials reported in earlier studies.^68–72^ The nearly 2.3-fold increase in specific surface area of the AC-ZnO composite compared with pristine ZnO further contributes to the enhanced electrolyte-accessible active sites and higher current response.

Moreover, pore size distribution was also analyzed for the AC, ZnO, and AC-ZnO composite (Fig. 3B). The AC, ZnO, and AC-ZnO composite exhibit average pore diameters of 1.37 nm, 1.73 nm, and 2.45 nm, respectively. The incorporation of ZnO into AC produced structural defects that improved the porosity of the AC-ZnO composite. The large surface area and porosity facilitate fast ion interactions with the electrode material surface and their diffusion during electrochemical performance. The enhanced specific surface area and porous structure of the AC-ZnO composite provide a larger number of electrochemically active sites and facilitate electrolyte-ion accessibility. In addition, the interconnected pore network promotes electrolyte penetration and shortens ion-diffusion pathways, leading to improved charge-transfer kinetics. Consequently, the AC-ZnO composite exhibits superior charge-storage capability and higher specific capacitance compared with the individual AC and ZnO electrodes.

Graphite sheets were selected as the current collector due to their high electrical conductivity, chemical stability, corrosion resistance, and excellent electrochemical performance.^73^ The graphitic surface contains edge defects and oxygen-containing functional groups generated during the cleaning process, which can act as favorable nucleation sites for ZnO deposition and promote strong interfacial adhesion between the active material and the substrate. Furthermore, the layered structure of graphite provides a large contact area and continuous electron-transport pathways, facilitating efficient charge transfer during electrochemical reactions. These characteristics not only improve the uniform distribution and mechanical stability of the deposited active material but also contribute to enhanced electrochemical performance and long-term cycling stability of the electrode.

### 3.3 XPS analysis

**Fig. 4:**
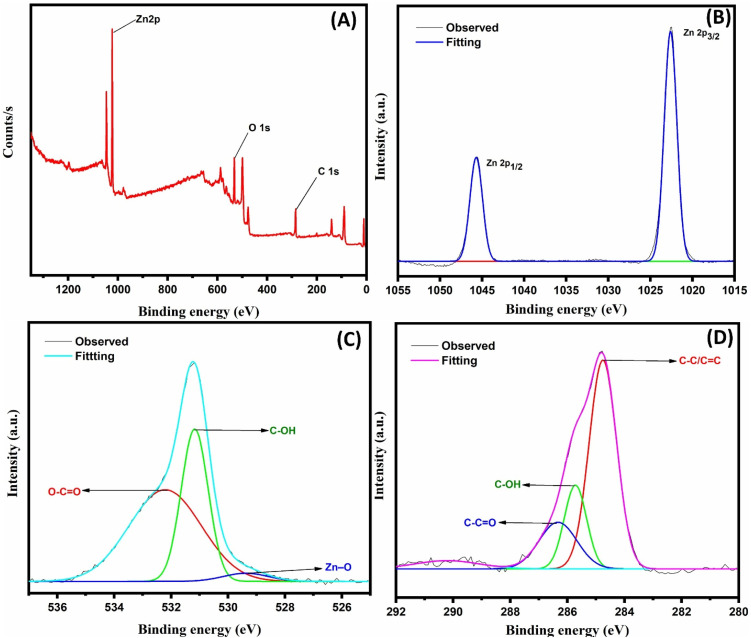
XPS spectrum; (A) full survey scan of AC-ZnO nanocomposites, (B) Zn 2p, (C) O 1s, and (D) C 1s of AC-ZnO nanocomposites.

The surface chemical composition and elemental states of the AC-ZnO composite were investigated by X-ray photoelectron spectroscopy (XPS), as shown in Fig. 4A–D. The survey spectrum (Fig. 4A) confirms the presence of Zn, O, and C elements in the composite, indicating the successful incorporation of ZnO into the activated carbon matrix.^74^ The high-resolution Zn 2p spectrum (Fig. 4B) exhibits two characteristic peaks located at approximately 1022.1 and 1045.2 eV, corresponding to Zn 2p_3/2_ and Zn 2p_1/2_, respectively.^75^ The spin–orbit splitting of about 23.1 eV confirms the presence of Zn^2+^ species, characteristic of ZnO. The high-resolution O 1s spectrum of the AC-ZnO composite (Fig. 4C) was deconvoluted into three distinct components located at 529.3, 531.2, and 532.2 eV. The peak at 529.3 eV is attributed to lattice oxygen (O^2−^) associated with the Zn–O bonds in the ZnO crystal structure.^76^ The dominant peak centered at 531.2 eV is assigned to oxygen-deficient regions and surface hydroxyl groups, indicating the presence of oxygen vacancies within the ZnO lattice. The peak at 532.2 eV corresponds to adsorbed oxygen species and oxygen-containing functional groups (such as C–O and O–C=O) present on the activated carbon surface. The presence of oxygen vacancies and surface oxygen functionalities can improve electrolyte wettability, facilitate ion transport, and provide additional electrochemically active sites, thereby enhancing the electrochemical performance of the AC-ZnO composite electrode. The high-resolution C 1s spectrum of the AC-ZnO composite (Fig. 4D) was deconvoluted into four components centered at 284.7, 285.7, 286.3, and 287.6 eV. The dominant peak at 284.7 eV is assigned to graphitic carbon (C–C/C=C), confirming the presence of a conductive carbon framework derived from activated carbon.^77^ The peaks at 285.7 and 286.3 eV correspond to C–O and C–OH functional groups, respectively, while the peak at 287.6 eV is attributed to carbonyl (C=O) species. The abundance of graphitic carbon provides efficient electron-transport pathways, whereas the oxygen-containing functional groups enhance electrolyte wettability and facilitate ion diffusion. These results confirm the successful formation of the AC-ZnO composite and indicate strong interfacial interactions between ZnO and the activated carbon matrix, which contribute to improved charge-transfer kinetics and enhanced electrochemical performance.

### 3.4 Electrochemical properties

**Fig. 5:**
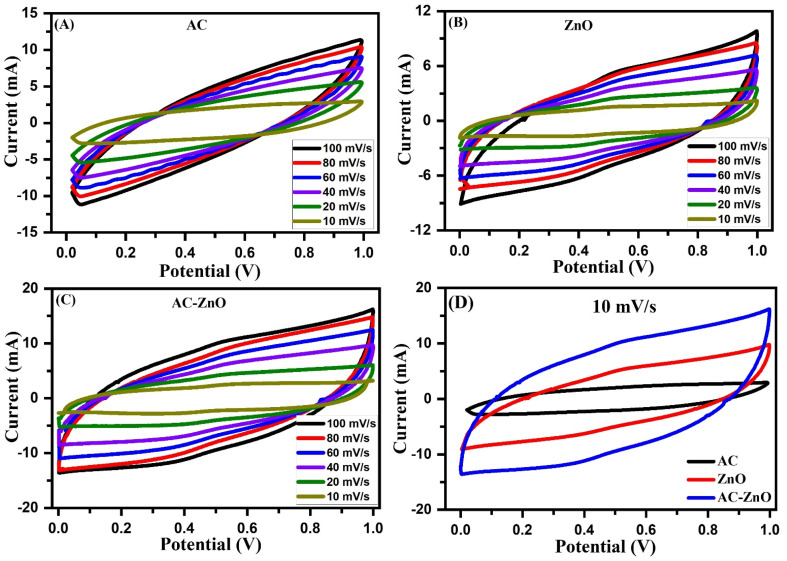
Electrochemical performance: CV curves of (A) AC, (B) ZnO, and (C) AC-ZnO, and (D) combined CV curves of AC, ZnO, and AC-ZnO composite at 10 mV s^−1^.

To evaluate the electrochemical performance of the as-prepared working electrodes, electrochemical tests including CV, GCD, and EIS were conducted using a three-electrode cell with 1 M H_2_SO_4_ aqueous solution as the electrolyte. Initially, CV curves were recorded at various scan rates of 10, 20, 40, 60, 80, and 100 mV s^−1^ within a voltage window of 0 to 1 V for AC, ZnO, and the AC-ZnO composite, as shown in Fig. 5. The potential window of 0–1.0 V was selected to maximize the charge-storage capability of the electrode while maintaining electrochemical stability. Although the electrochemical response appears favorable near 0.9 V, the CV and GCD curves remain stable and nearly symmetric up to 1.0 V without noticeable distortion or significant polarization. Therefore, 1.0 V was considered the optimum operating potential window for evaluating the electrochemical performance of the AC-ZnO electrode.

Fig. 5A displays the CV curves of the AC electrode, which exhibit a quasi-rectangular shape. The symmetric anodic and cathodic branches indicate the electrode's capacitive behavior due to the electrical double layer. The AC electrode stores charge through electrostatic adsorption–desorption of ions at the electrode–electrolyte interface.^78,79^ The increasing area and current of the CV curves with higher scan rates demonstrate good rate capability and fast ion kinetics at the porous AC surface.^74^Fig. 5B displays the CV curves of ZnO at various scan rates from 10 to 100 mV s^−1^. The distorted rectangular shape and minor humps indicate a combination of electrical double-layer and pseudocapacitive behavior of the ZnO electrode. ZnO exhibits a high current response and larger CV area compared to AC due to its faradaic redox reactions; however, at higher scan rates, the rectangular shape becomes slightly distorted, and the current response declines because of increased charge transfer resistance and limited ion diffusion. Particle agglomeration and the semiconducting properties of ZnO contribute to reduced electrochemical performance at high scan rates.^80–82^ The AC-ZnO electrode shows a larger current response and CV area compared to AC and ZnO alone, confirming enhanced capacitive properties (Fig. 5C). The CV curves are symmetric, and the current response gradually increases with higher scan rates, indicating excellent rate capability, reversibility, and low internal resistance in the AC-ZnO electrode. The synergistic effects between AC and ZnO improve the electrochemical performance of the composite electrode. The AC-ZnO electrode exhibits excellent electrochemical behavior due to the high electrical conductivity of AC and the rapid redox reactions of ZnO.^83^

Additionally, the uniform dispersion of ZnO on the AC surface, as confirmed by SEM and TEM analyses, ensures efficient utilization of active sites and prevents significant ZnO agglomeration. Fig. 5D displays the combined CV curves of AC, ZnO, and AC-ZnO at a constant scan rate of 10 mV s^−1^. The AC-ZnO composite exhibits a high current response and a large CV area, indicating improved electrochemical performance relative to ZnO and AC. The smallest enclosed area in the current response is observed for AC compared to ZnO and the AC-ZnO composite, indicating pure electrical double-layer capacitive behavior. These results confirm that combining ZnO with AC effectively enhances the electrochemical kinetic process and overall capacitive properties.

The significant increase in the current density window observed for the AC-ZnO composite electrode (Fig. 5) can be attributed to the synergistic interaction between activated carbon (AC) and ZnO. Activated carbon provides a highly porous framework with a large specific surface area and excellent electrical conductivity, facilitating rapid electron transport and efficient electrolyte ion diffusion throughout the electrode. Simultaneously, ZnO contributes additional electrochemically active sites and enhances charge storage through pseudocapacitive behavior arising from reversible faradaic redox reactions. The combined effect of the electrical double-layer capacitance of AC and the pseudocapacitance of ZnO results in improved charge-transfer kinetics, enhanced ion accessibility, and superior electrochemical performance, thereby enabling the AC-ZnO composite electrode to operate effectively over a wider current density range. The integration of ZnO with the conductive activated carbon (AC) matrix enhances charge-transfer kinetics and increases the number of electrochemically accessible active sites for charge storage. Furthermore, the porous structure of AC facilitates efficient electrolyte penetration and ion diffusion, while ZnO provides additional charge-storage capability through reversible faradaic redox reactions. Consequently, the AC-ZnO composite exhibits a larger electrochemically active surface area, improved electrical conductivity, and enhanced redox activity compared to the individual AC and ZnO electrodes. These synergistic effects contribute to a substantially higher current response and an expanded current density window in the CV curves.

**Fig. 6:**
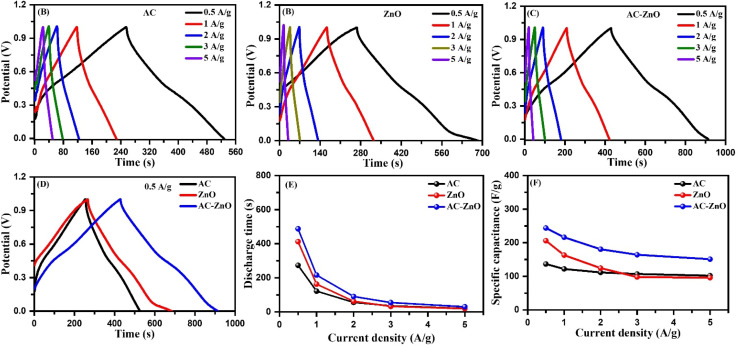
GCD curves of (A) AC, (B) ZnO, and (C) AC-ZnO, and (D) combined GCD curves at 0.5 A g^−1^, (E) discharge time, and (F) *C*_s_ of AC, ZnO, and AC-ZnO composite at 0.5, 1, 2, 3, and 5 A g^−1^.

The GCD curves of AC, ZnO, and AC-ZnO composite electrodes were measured at different current densities, including 0.5, 1, 2, 3, and 5 A g^−1^, as shown in Fig. 5. The GCD curves for AC are symmetric and triangular, indicating electrical double-layer capacitive behavior (Fig. 6A). The linear relationship between time and voltage, along with negligible IR drops, demonstrates the remarkable reversibility and rate capability of the AC electrode, which has minimal internal resistance. However, the discharge time is short due to the electrical double-layer capacitive process at the AC surface.^84^ The ZnO electrode displays non-linear GCD curves, indicating faradaic redox reactions occurring at its surface (Fig. 6B). ZnO exhibits a longer discharge time than AC, reflecting higher capacitance from pseudocapacitive behavior in addition to electrical double-layer capacitance. Nevertheless, limited ion diffusion leads to polarization and nanoparticle (NP) agglomeration, which reduces charge storage at high current densities.^84^ The formation of a composite between ZnO and AC increases the discharge time (Fig. 6C). The GCD curves become increasingly symmetric and exhibit longer discharge times with increasing current density. However, the curves slightly deviate from an ideal triangle shape, indicating a combination of electrical double-layer and pseudocapacitive behavior in the AC-ZnO electrode. The longer discharge time correlates with higher specific capacitance for AC-ZnO, arising from the synergistic effects of AC and ZnO NPs. As previously mentioned, AC provides a conductive framework, whereas ZnO contributes pseudocapacitive charge storage through reversible faradaic reactions, contributing to excellent electrochemical performance. The close interfacial contact between AC and ZnO facilitates rapid electron transport and efficient electrolyte ion diffusion, thereby enhancing electrochemical kinetics. Fig. 6D clearly compares the GCD curves of AC, ZnO, and AC-ZnO electrodes at a constant current density of 0.5 A g^−1^. The AC-ZnO shows a significantly longer discharge time compared to the other two electrodes (Fig. 6E), indicating superior electrochemical performance. The IR drop is also lower in AC-ZnO, thanks to the conductive AC matrix. A small IR drop is observed at the charge–discharge transition near the upper potential limit. This voltage loss originates from the internal resistance of the electrochemical system, including the solution resistance, charge-transfer resistance, and electrode/current-collector contact resistance. The relatively small IR drop of the AC-ZnO electrode indicates efficient electron transport and rapid charge-transfer kinetics, which is consistent with the low resistance values obtained from EIS analysis. The reduced polarization contributes to the superior capacitive performance and excellent rate capability of the composite electrode. Discharge time is maximum at 0.5 A g^−1^ and gradually decreases as current density increases to 5 A g^−1^ (Fig. 6E) for all electrodes. The reduced discharge time at higher current densities results from limited ion–electrode interactions. Notably, ZnO has a shorter discharge time compared to AC and AC-ZnO, due to nanoparticle agglomeration and fewer active sites at the maximum current density. Fig. 6F shows the specific capacitance of AC, ZnO, and AC-ZnO electrodes at different current densities. Fig. S1 presents the specific capacitance values of AC, ZnO, and AC-ZnO electrodes from independent measurements at a current density of 0.5 A g^−1^. The pristine AC electrode exhibited a specific capacitance of 136 ± 5.57 F g^−1^, whereas the ZnO electrode delivered a higher capacitance of 203.3 ± 4.04 F g^−1^. Notably, the AC-ZnO composite electrode achieved the highest specific capacitance of 239 ± 4.58 F g^−1^. The gradual decrease in specific capacitance with increasing current density is due to limited ion diffusion and incomplete active site-ion interactions.^85^ The high specific capacitance of the AC-ZnO electrode confirms its better rate capability, thanks to the porous structure of AC that facilitates fast ion transport and the pseudocapacitive contribution of ZnO through reversible redox reactions. Overall, the electrochemical performance of AC-ZnO surpasses that of other similar materials listed in Table 2.

**Table 2:** Comparative electrochemical performance

Material	*C*_s_ (F g^−1^)	Current density (A g^−1^)	Electrolyte	Method	Reference
ZnO/carbon	141	0.1	1 M KOH	GCD	86
ZnO/activated carbon	198	1	6 M KOH	GCD	87
AC-ZnO	210	1	1 M KOH	GCD	88
ZnO/CNT	98	1	1 M Na_2_SO_4_	GCD	89
Nd-doped ZnO	154	2.5	1 M KCl	GCD	90
AC-ZnO	243.86	0.5	1 M H_2_SO_4_	GCD	This work

**Fig. 7:**
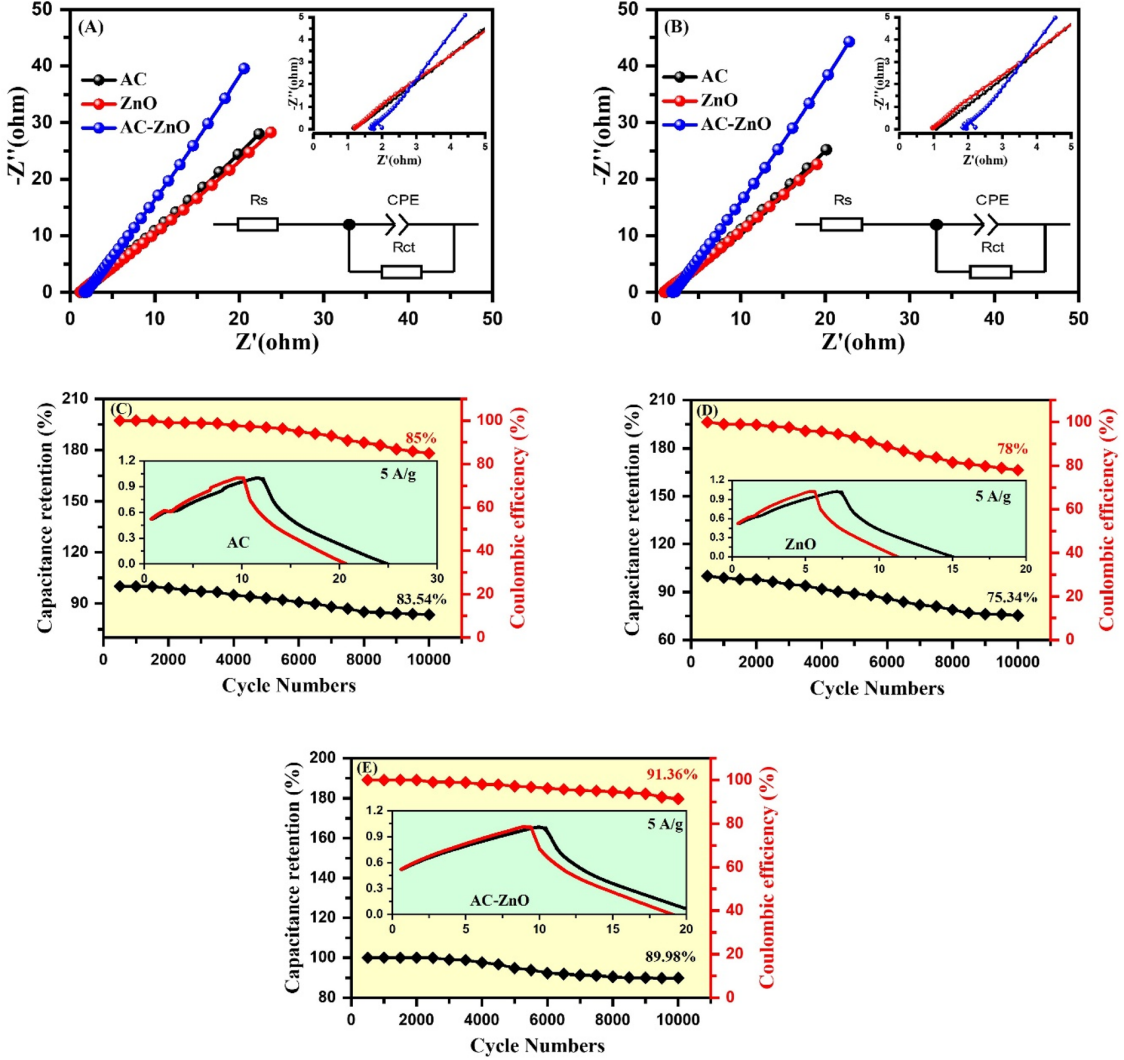
EIS curves of AC, ZnO, and AC-ZnO electrodes: (A) before 10 000 cycles, (B) after 10 000 cycles; capacitance retention and coulombic efficiency of (C) AC, (D) ZnO, and (E) AC-ZnO electrodes over 10 000 cycles analyzed at 5 A g^−1^.

Fig. 7 presents the EIS curves of AC, ZnO, and the AC-ZnO composite electrode. The EIS data were fitted using *Z*-view software, with the corresponding electronic circuit shown in the inset images of Fig. 7A and B for before and after 10 000 cycles. The intercept in the high-frequency region corresponds to the solution resistance (*R*_s_), indicating the overall resistance of the electrochemical system. The semicircle in the high-frequency region represents the charge-transfer resistance (*R*_ct_) at the electrode–electrolyte interface, whereas the linear region at low frequencies is associated with the diffusion of electrolyte ions within the electrode structure.^91^ The cycling stability test was conducted at a current density of 5 A g^−1^ to evaluate the long-term durability of the electrode under high-rate charge–discharge conditions. Stability testing at higher current densities provides a more rigorous assessment of structural integrity, electrochemical reversibility, and ion-transport stability during prolonged cycling. Therefore, the cycling performance at 5 A g^−1^ offers a reliable indication of the practical durability of the AC-ZnO electrode. All electrodes show a negligible semicircle diameter, suggesting very low *R*_ct_ before and after 10 000 cycles (Fig. 7A and B). The *R*_ct_ values for the AC, ZnO, and AC-ZnO electrodes are very low, namely 0.05 or 0.054 Ω, 0.061 or 0.068 ohm, and 0.035 or 0.036 ohm, respectively, before and after 10 000 cycles (Fig. 7A and B).

The AC-ZnO electrode exhibits a lower *R*_ct_ due to the synergistic effects between AC and ZnO NPs, along with rapid ion interactions at defect-rich active sites.^92^ The intercepts on the EIS curves are 1.64, 1.78, and 1.32 ohms, representing the equivalent series resistance (*R*_s_) of AC, ZnO, and AC-ZnO electrodes before 10 000 cycles. After 10 000 cycles, *R*_s_ slightly increases to 1.72, 1.88, and 1.34 ohms. The Warburg diffusion curve indicates the ion diffusion rate on the electrode surface, with slope values of 1.28, 1.22, and 2.04 for AC, ZnO, and AC-ZnO, confirming that enhanced ion diffusion occurs in the composite electrode due to its porous structure. The shift of the Nyquist plot toward lower *X*-axis intercept values for the AC-ZnO electrode indicates a reduction in internal resistance and improved charge-transfer kinetics resulting from the synergistic interaction between AC and ZnO. The lower *R*_s_ and *R*_ct_ values of the composite electrode confirm enhanced electrical conductivity and faster electron transfer at the electrode/electrolyte interface. Furthermore, the steeper low-frequency slope suggests improved ion diffusion and more ideal capacitive behavior, which collectively contribute to the superior electrochemical performance of the AC-ZnO electrode. After 10 000 cycles, the slope of AC and ZnO decreases to 1.24 and 1.16, respectively, while the slope of AC-ZnO increases to 2.12, demonstrating that the composite electrode's excellent capacitive performance results from enhanced ion diffusion.

The cycling stability and specific capacitance retention are crucial for the practical use of the prepared electrode in energy storage devices. Fig. 7C–E illustrates the specific capacitance retention and coulombic efficiency of the AC, ZnO, and AC-ZnO electrodes over 10 000 charge–discharge cycles. Among the investigated electrodes, the AC-ZnO electrode exhibits superior cycling stability, retaining 89.98% of its initial specific capacitance with a coulombic efficiency of 91.36% after 10 000 cycles. In comparison, the ZnO and AC electrodes retain 75.34% and 83.54% of their initial specific capacitance, respectively, corresponding to coulombic efficiencies of 78% and 75% after 10 000 cycles. These results demonstrate the enhanced electrochemical stability and reversibility of the AC-ZnO composite electrode.

The excellent cycling stability of the AC-ZnO electrode can be attributed to the high electrical conductivity of AC and the rapid redox reactions of ZnO. In addition, the high surface area and porous structure facilitate enhanced ion transport and provide abundant active sites for ion interactions, resulting in superior electrochemical performance during prolonged cycling. It is important to note that the relatively low capacitance retention and coulombic efficiency of the ZnO electrode are primarily due to particle agglomeration and restricted ion diffusion during long-term cycling. Furthermore, rapid redox reactions may lead to a gradual reduction in the number of accessible active sites for ion interactions during repeated charge–discharge cycles. In contrast, the synergistic combination of electrical double-layer capacitance and pseudocapacitive behavior enables the AC-ZnO electrode to exhibit outstanding cycling stability, maintaining excellent electrochemical performance even after 10 000 cycles at a current density of 5 A g^−1^.

The excellent cycle life of the AC-ZnO electrode is due to the high electrical conductivity of AC and rapid redox reactions in ZnO. The maximum surface area and porous structure support optimal ion interactions and charge storage in the composite electrode, showing excellent performance during long cyclic processes. The low capacitance retention and coulombic efficiency of the ZnO electrode are due to nanoparticle agglomeration and small pores, which limit ion diffusion during extended cycling. Rapid redox reactions reduce the active sites available for ion interactions during electrochemical performance. However, the combination of electrical double-layer capacitive behavior and pseudocapacitive behavior allows the AC-ZnO electrode to demonstrate superior cycle life, even after 10 000 cycles at 5 A g^−1^. SEM images of the AC-ZnO electrode after 10 000 cycles (Fig. S2) confirm the structural stability of the electrode materials.

### 3.5 Kinetic analysis using Dunn's model

The charge storage behavior and kinetics can be examined from CV curves by performing calculations using Dunn's model equations.^83,84^

8 *i* = *av*^*b*^

**Fig. 8:**
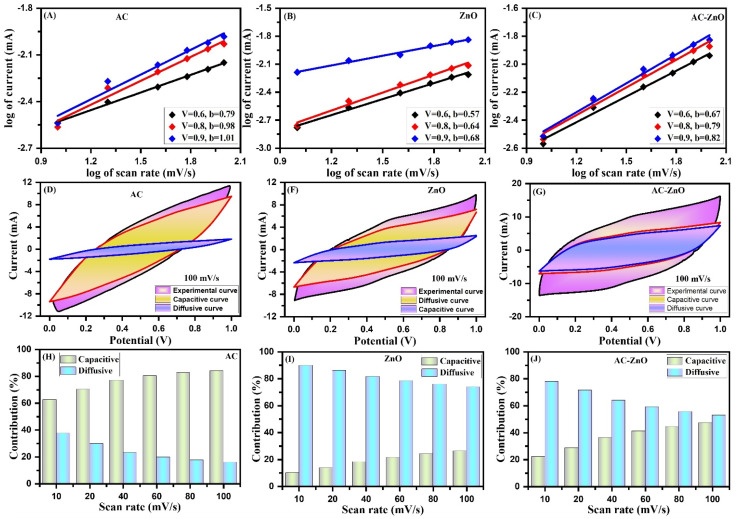
Dunn's power law simulated results: calculated *b* values of (A) AC, (B) ZnO, and (C) AC-ZnO, capacitive–diffusive controlled curves of (D) AC, (E) ZnO, and (F) AC-ZnO at 100 mV s^−1^, and capacitive–diffusive controlled contributions of (G) AC, (H) ZnO, and (I) AC-ZnO at different scan rates.

9 *i*(*V*) = *k*_1_*v* + *k*_2_*v*^1/2^

where *k*_1_ and *k*_2_ are variable parameters and *i* represents the current response at different scan rates (*v*). The *b* value is determined from the slope of the eqn (8) (log(*i*) *versus* log(*v*)) plot by observing the current at a fixed potential for various scan rates, as shown in Fig. 8A–C. Typically, the electrode displays electrical double-layer capacitive behavior if the *b* value is one, while the *ab* value of 0.5 indicates pseudocapacitive behavior. Values between 0.5 and 1 suggest hybrid supercapacitive behavior.^93^ The AC electrode exhibits maximum *b* values of 0.79, 0.98, and 1.01 at 0.6, 0.8, and 0.9 V, confirming electrical double-layer capacitive behavior (Fig. 8A). The ZnO electrode shows pseudocapacitive behavior, with *b* values of 0.57, 0.64, and 0.68 at these voltages (Fig. 8B). Interestingly, the AC-ZnO composite displays hybrid supercapacitor behavior, with *b* values of 0.67, 0.79, and 0.82 at 0.6, 0.8, and 0.9 V (Fig. 8C). This hybrid behavior results from the electrical double-layer capacitance of AC alongside pseudocapacitance from ZnO. The hybrid charge-storage behavior of the AC-ZnO composite arises from the synergistic combination of electrical double-layer capacitance (EDLC) and pseudocapacitance.^94^ The porous activated carbon framework provides a high surface area and a conductive network for rapid electrolyte-ion adsorption and electron transport, resulting in dominant capacitive-controlled charge storage. Meanwhile, ZnO contributes additional charge storage through reversible faradaic redox reactions, which account for the diffusion-controlled component observed in Dunn's analysis. Consequently, the coexistence of these two charge-storage mechanisms enables the AC-ZnO electrode to exhibit enhanced electrochemical performance and hybrid supercapacitive behavior.

Moreover, the electrical double layer and pseudocapacitive behavior curves and contributions (due to surface-controlled and diffusion-controlled processes) can also be simulated using the following equations.^95^10
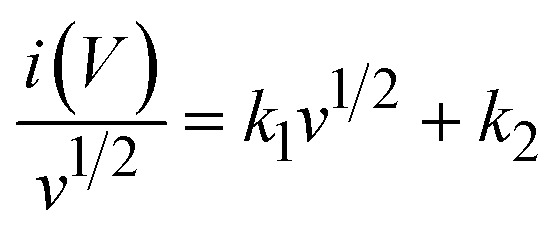
where *k*_1_ and *k*_2_ parameters correspond to surface-controlled (capacitive) and diffusion-controlled (diffusive) contributions acquired from the slope and intercept of the plot of 
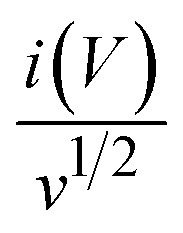
*versus v*^1/2^. The diffusive and capacitive currents collectively contribute to generating the total current response of said electrode. Therefore, these currents should be significant to produce a large current by said electrode at the specified scan rate. Fig. 8D–F shows that the experimental curve is contributed by the diffusive and capacitive curves of AC, ZnO, and AC-ZnO electrodes measured at 100 mV s^−1^, respectively. The AC shows the maximum capacitive current response as compared to ZnO and AC-ZnO. The ZnO shows a larger diffusive current response, whereas AC-ZnO exhibits approximately similar capacitive and diffusive current responses. The presence of oxygen causes a diffusion process in ZnO and the AC-ZnO electrode. The AC-ZnO electrode shows hybrid behavior due to the electrical double-layer capacitive behavior of AC and pseudocapacitive behavior of ZnO.

Fig. 8H–J shows the diffusive and capacitive contributions for AC, ZnO, and AC-ZnO electrodes at various scan rates: 10, 20, 40, 60, 80, and 100 mV s^−1^. The AC displays higher capacitive contributions of 62.53, 70.24, 76.95, 80.34, 82.52, and 84.07%, compared to ZnO (10.07, 13.67, 18.29, 21.52, 24.05, and 26.15%) and AC-ZnO (22.06, 28.58, 36.15, 40.94, 44.46, and 47.23%) at these scan rates. The maximum capacitive contributions in AC are due to small pores and high electrical conductivity. The formation of composites between AC and ZnO introduces more structural defects, a larger surface area, and a porous structure, leading to improved performance of the AC-ZnO electrode. The increasing capacitive behavior indicates excellent rate capability, rapid charge transfer, and storage capacity of the optimal sample.

### 3.6 Asymmetric device performance

**Fig. 9:**
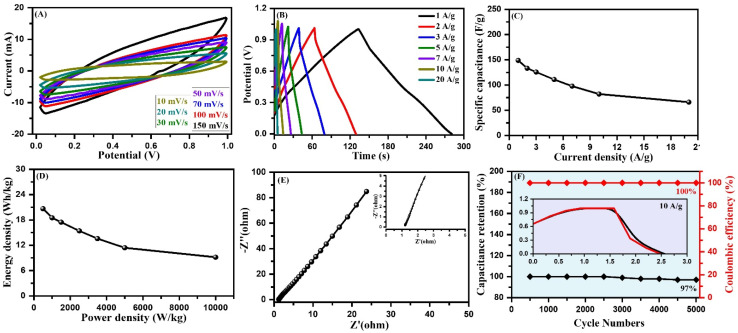
Asymmetric supercapacitor device performance of the AC-ZnO electrode: (A) CV curves (10–150 mV s^−1^); (B) GCD curves; (C) *C*_s_; (D) Ragone plots; (E) Nyquist plot, and (F) cycling performance.

The AC-ZnO sample was used to assemble an asymmetric device with a 1 M H_2_SO_4_ electrolyte to evaluate its performance in energy storage applications. First, CV curves were recorded at 10, 20, 30, 50, 70, 100, and 150 mV s^−1^ within a potential range of 0–1 V (Fig. 9A) to analyze its capacitive behavior. The CV curves are nearly rectangular at all scan rates, indicating excellent stability, rate capability, reversibility, and superior capacitive performance of the assembled device. Similarly, GCD curves were obtained at different current densities of 1, 2, 3, 5, 7, 10, and 20 A g^−1^ (Fig. 9B). The triangular symmetric shape persists across all current densities, confirming the EDLC behavior of the asymmetric device. The *C*_s_ values of the assembled device are 149, 133, 126, 111, 98, 82, and 66 F g^−1^ at 1, 2, 3, 5, 7, 10, and 20 A g^−1^, respectively (Fig. 9C). The discharge time gradually decreases with increasing current density due to limited ion adsorption at the electrode surface. At higher current densities, ion diffusion is restricted and ion penetration decreases, leading to lower *C*_s_ (specific capacitance) values. Additionally, *E*_d_ and *P*_d_ are critical parameters for assessing the performance of the asymmetric device in practical applications. A maximum *E*_d_ of 20.66 Wh kg^−1^ and a minimum *P*_d_ of 500 W kg^−1^ are observed for the device (Fig. 9D).

The superior electrochemical performance is due to high surface area, maximum porosity, and a hierarchical network that provides effective pathways for rapid ion movement at the AC-ZnO electrode. The solution resistance (*R*_s_), charge transfer resistance (*R*_ct_), and ion diffusion rate within the assembled device were also examined through EIS analysis, covering a frequency range from 0.01 Hz to 100 kHz (Fig. 9E). The presence of a nearly vertical EIS curve confirms ideal capacitive behavior. A small semicircle in the high-frequency region indicates a low *R*_ct_ of 0.06 ohms, confirming fast ion movement at the electrode–electrolyte interface. The *R*_s_ is 0.95 ohms, with a Warburg diffusion slope of 4.45, showing low resistance due to the high electrical conductivity of the AC-ZnO electrode. To verify these electrochemical results, a long-term cycling stability test was performed for 5000 cycles (Fig. 9F). The device retained 97% of its *C*_s_ and showed a coulombic efficiency of 100%, demonstrating excellent reversibility and stability.

The superior electrochemical performance of the AC-ZnO composite is closely related to its structural and morphological characteristics. The incorporation of ZnO into the activated carbon matrix generates additional electrochemically active sites, while the porous and interconnected structure facilitates electrolyte penetration and ion transport. Moreover, the increased specific surface area and pore volume enhance electrolyte accessibility and charge-storage capability. These features collectively promote efficient electron transport, faster ion diffusion, and improved charge-transfer kinetics, resulting in higher specific capacitance, lower internal resistance, enhanced rate capability, and excellent cycling stability of the AC-ZnO electrode.

## 4. Conclusion

In conclusion, activated carbon was produced from rice straw waste and then combined with ZnO nanoparticles prepared *via* a hydrothermal method. The materials were characterized using XRD, Raman, FTIR, BET, SEM, and TEM to analyze their crystal structure, vibrational modes, functional groups, surface area, surface morphology, and microstructures, respectively. The peak intensity, crystallite size, and *d*-spacing decreased due to the formation of the AC-ZnO composite. The AC-ZnO composite exhibited a higher specific surface area of 578 m^2^ g^−1^ compared with 201 and 255 m^2^ g^−1^ for AC and ZnO, respectively. It achieved a maximum specific capacitance of 244 F g^−1^ at 0.5 A g^−1^. The AC, ZnO, and AC-ZnO retained 83.54%, 75.34%, and 89.98% of their initial capacitance after 10 000 cycles. The AC-ZnO displayed hybrid supercapacitive behavior. The assembled device demonstrated a specific capacitance of 149 F g^−1^, energy density of 20.66 Wh kg^−1^, and power density of 500 W kg^−1^ at 1 A g^−1^, with 97% capacitance retention and 100% coulombic efficiency after 5000 cycles. These results demonstrate that the AC-ZnO nanocomposite is a promising electrode material for high-performance supercapacitor applications.

## Conflicts of interest

The authors declare that they have no known competing financial interests or personal relationships that could have appeared to influence the research work reported in the manuscript.

## Supplementary Material

NA-OLF-D6NA00276E-s001

## Data Availability

The collected results related to the manuscript will be made available upon reasonable request to the corresponding author. The data supporting this article have been included as part of the supplementary information (SI). Supplementary information is available. See DOI: https://doi.org/10.1039/d6na00276e.
