# Supplementary material for: Rice straw-derived activated carbon/ZnO nanocomposite as a high-performance electrode for asymmetric supercapacitors
Source: Nanoscale Adv. 2026 Jul 9;8(16):4656–71. doi: 10.1039/d6na00276e (PMC13400085; doi:10.1039/d6na00276e)
Supplement: Supplementary file 1 [file NA-008-D6NA00276E-s001.pdf]

Supplementary Information (SI) for Nanoscale Advances.
This journal is © The Royal Society of Chemistry 2026

Rice Straw-Derived Activated Carbon/ZnO Nanocomposite as a High-Performance Electrode for Asymmetric Supercapacitors

Asif Raza[a], Pravin MBS[b], Divya Rajendran[c], Zartasha Sarwar[d], Ranjithkumar Ravi[e], Nguyen Xuan Sang[f, g], Subramanian Ramanathan[f, h*]

[a]Department of Physics, University of South Africa, Africa.

[b]Department of Chemical Engineering and Materials Science, Amrita University, Coimbatore, India.

[c]Medical Imaging Key Laboratory of Sichuan Province, Department of Oncology, Affiliated Hospital of North Sichuan Medical College, Nanchong, Sichuan, China.

[d]Department of Physics, The Women University Multan, Pakistan.

[e]Department of Physics, Faculty of Arts, Science, Commerce & Management, Karpagam Academy of Higher Education (Deemed to be University), Coimbatore, India.

[f]Atomic Molecular and Optical Physics Research Group, Institute for Advanced Study in Technology, Ton Duc Thang University, Ho Chi Minh City, Vietnam.

[g]Faculty of Electrical and Electronics Engineering, Ton Duc Thang University, Ho Chi Minh City, Vietnam

[h]Faculty of Applied Sciences, Ton Duc Thang University, Ho Chi Minh City, Vietnam

[*]Corresponding author: Subramanian Ramanathan, Email: subramanianramanathan@tdtu.edu.vn

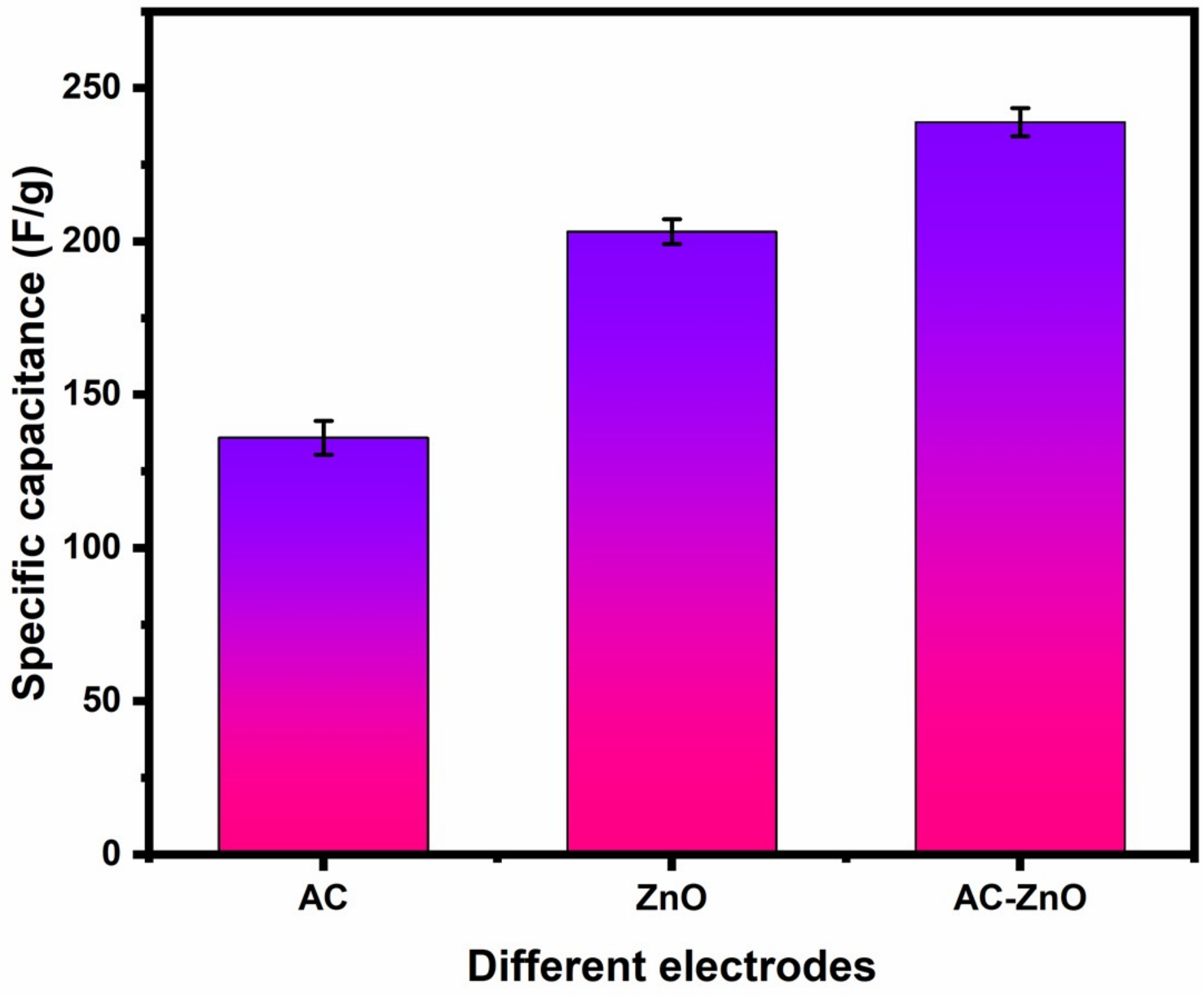

Figure S1: Specific capacitance of AC, ZnO, and AC-ZnO electrodes at 0.5 A g^{-1} current density.

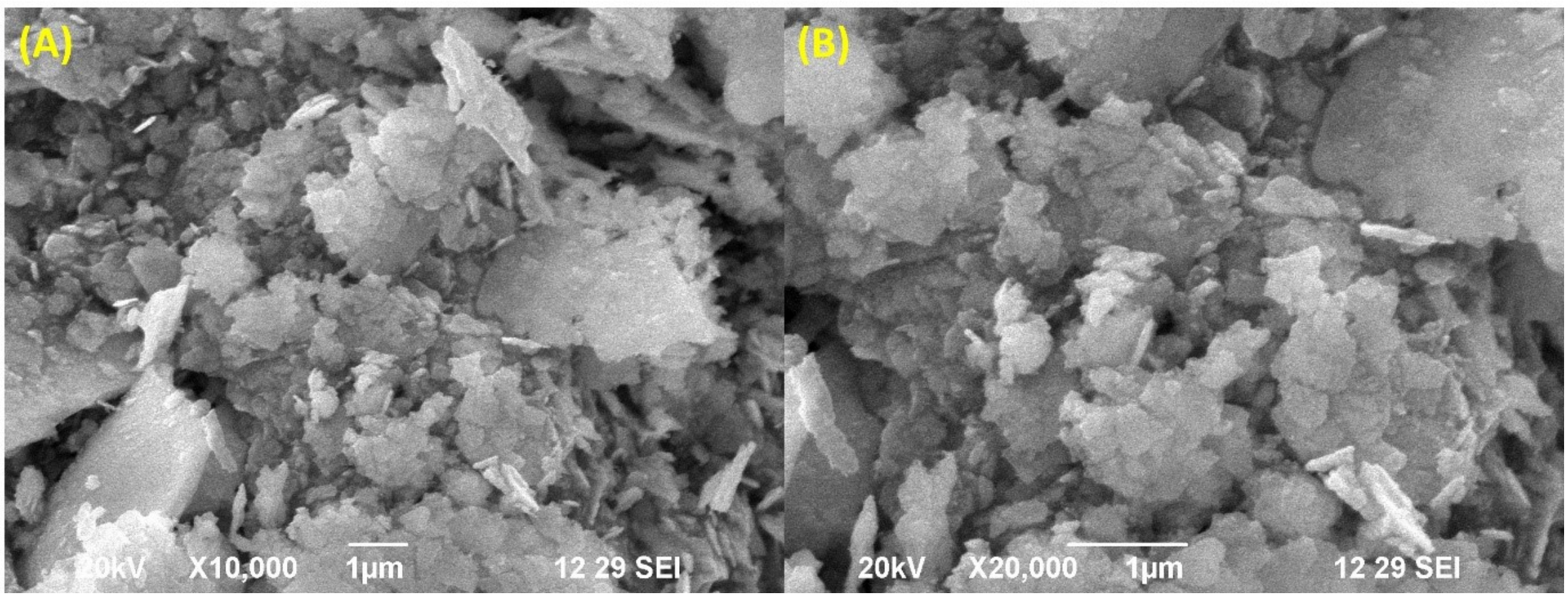

Figure S2: SEM images of AC/ZnO electrode materials after 10000 cycles (A-B).
